# Habitat Segregation Patterns of Container Breeding Mosquitos: The Role of Urban Heat Islands, Vegetation Cover, and Income Disparity in Cemeteries of New Orleans

**DOI:** 10.3390/ijerph19010245

**Published:** 2021-12-26

**Authors:** Rebeca de Jesús Crespo, Rachel Elba Rogers

**Affiliations:** Department of Environmental Sciences, Louisiana State University, Baton Rouge, LA 70803, USA; rerogers2020@gmail.com

**Keywords:** *Aedes aegypti*, *Aedes albopictus*, urban heat island (UHI), income disparity vegetation cover, cemeteries, New Orleans

## Abstract

*Aedes aegypti* and *Aedes albopictus* are important pathogen-carrying vectors that broadly exhibit similar habitat suitability, but that differ at fine spatial scales in terms of competitive advantage and tolerance to urban driven environmental parameters. This study evaluated how spatial and temporal patterns drive the assemblages of these competing species in cemeteries of New Orleans, LA, applying indicators of climatic variability, vegetation, and heat that may drive habitat selection at multiple scales. We found that *Ae. aegypti* was well predicted by urban heat islands (UHI) at the cemetery scale and by canopy cover directly above the cemetery vase. As predicted, UHI positively correlate to *Ae. aegypti*, but contrary to predictions, *Ae. aegypti*, was more often found under the canopy of trees in high heat cemeteries. *Ae. albopictus* was most often found in low heat cemeteries, but this relationship was not statistically significant, and their overall abundances in the city were lower than *Ae. aegypti*. *Culex quinquefasciatus*, another important disease vector, was also an abundant mosquito species during the sampling year, but we found that it was temporally segregated from *Aedes* species, showing a negative association to the climatic variables of maximum and minimum temperature, and these factors positively correlated to its more direct competitor *Ae. albopictus*. These findings help us understand the mechanism by which these three important vectors segregate both spatially and temporally across the city. Our study found that UHI at the cemetery scale was highly predictive of *Ae. aegypti* and strongly correlated to income level, with low-income cemeteries having higher UHI levels. Therefore, the effect of excessive heat, and the proliferation of the highly competent mosquito vector, *Ae. aegypti*, may represent an unequal disease burden for low-income neighborhoods of New Orleans that should be explored further. Our study highlights the importance of considering socioeconomic aspects as indirectly shaping spatial segregation dynamics of urban mosquito species.

## 1. Introduction

The container-breeding mosquitos, *Aedes aegypti* and *Aedes albopictus*, are important pathogen-carrying vectors that thrive in urban environments. Although these mosquitos may share habitat suitability broadly, at fine spatial scales they have different levels of tolerance to urban driven environmental changes and differing competitive advantages [1,2]. *Aedes albopictus* is thought to be a superior competitor due to its ability to better exploit food resources, such as leaf litter within container habitats at the larval stage [1,2,3]. This competitive advantage has been linked to a displacement of *Ae. aegypti*, from urban areas across the United States, where *Ae. albopictus* has been introduced [2]. *Aedes aegypti’s* competitive inferiority may be alleviated by its higher ability to tolerate heat and egg desiccation [1,2], which may be especially common within cities due to urban heat island (UHI) effects. This interplay between competitive ability to exploit resources and the ability to disperse across urban-heat island patches may allow for the coexistence of these two species in certain places, rather than complete competitive exclusion [2,4].

The ability of container mosquitoes to segregate spatially across a city’s landscape is also influenced by the actual availability of container habitats where mosquito larvae can develop. Container mosquitos often exploit human made items such as automobile tires, flowerpots, and other discarded water holding vessels. Container habitat availability is in turn influenced by a variety of socioeconomic factors. In the USA, studies have shown that container mosquitos and their habitats increase proportionally with abandoned properties, urban disinvestment, and waste mismanagement, all of which are factors that disproportionally affect low-income neighborhoods [5,6,7,8]. This highlights the importance of considering heterogeneity in the social dimension of the urban landscape when evaluating mosquito vector community assemblages.

An underlying factor jointly associated with socioeconomic disparities and urban heat islands is that of urban vegetation cover. Within a city, vegetation provides a cooling effect by means of shading and evapotranspiration, and this effect has been shown consistently to mitigate UHI [9]. However, the distribution of vegetation can be affected by factors such as “the luxury effect”, wherein higher income neighborhoods may be better able to afford green space management, historical urban legacies, which may vary by context, and education level, which links to knowledge and perceptions about ecosystem services [10]. Important ecosystem services provided by urban vegetation, such as shading, air purification, and improved neighborhood aesthetics, have been linked to health factors such as higher physical activity, lower cardiovascular disease, and improved mental health [11,12]. However, few studies have linked vegetation cover to ecosystem services that affect the dynamics of mosquito-borne diseases [13].

In the context of the coexistence of *Ae. albopictus* and *Ae. aegypti*, vegetation driven differences in habitat selection due to food sources or microhabitat qualities may be relevant for human health. *Aedes albopictus*, which is often associated with more vegetated and cooler habitats [2] and which is better able to exploit leaf litter as a food source [1,2,3], is also a less effective vector for one of the most prevalent mosquito borne disease, dengue fever. This is due partly to its lower infectivity rates to certain dengue virus serotypes [14], and partly due to its higher frequency of feeding on non-human hosts [15]. Therefore, vegetation cover in a city could be thought of as providing an indirect ecosystem service that limits the presence of the most competent mosquito vector, *Ae. aegypti*. Conversely, UHI, and climate change may have the opposite effect.

In this study, we evaluate linkages between temporal abiotic factors (rainfall, temperature), landscape level factors (UHI, vegetation cover, and income disparities), and microhabitat level factors (canopy cover) to the abundance patterns of *Ae. aegypti* and *Ae. albopictus* larvae in cemeteries of New Orleans, Louisiana. Our study approach used a multi-scale habitat evaluation (see graphical abstract), where abiotic drivers are contextualized with aspects of importance to spatial segregation at the cemetery scale, the container scale and temporally. We hypothesize that the two species coincide temporally in relation to rainfall and temperature suggesting overlap in their abiotic requirements in New Orleans. We hypothesize that *Ae. albopictus’* sensitivity to heat limits its presence in un-vegetated heat islands within the city. At the micro-scale (cemetery vases), where larval competition is likely to occur, we hypothesize that canopy cover from trees would favor *Ae. albopictus* and exclude *Ae. aegypti*, due to the latter’s competitive inferiority in exploiting resources, particularly leaf litter.

## 2. Materials and Methods

### 2.1. Study Site

**Figure 1:**
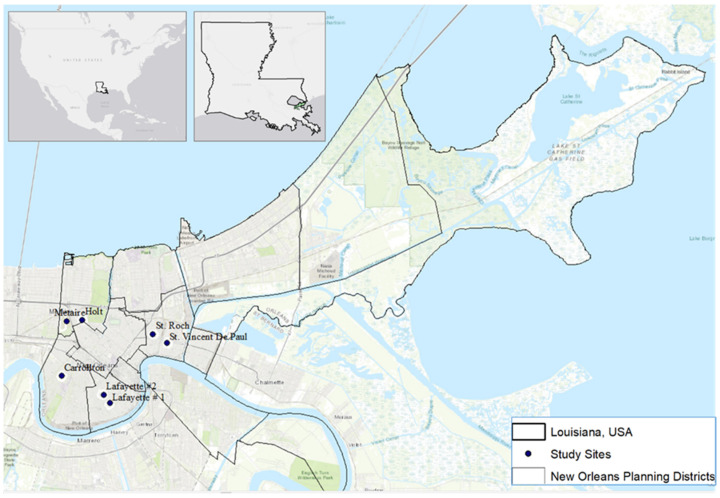
Study sites in the city of New Orleans, LA, located in the Southeastern USA. Topographic map source: Esri, Garmin, Intermap, increment P Corp., GEBCO, USGS, FAO, NPS, NRCAN, GeoBase, IGN, Kadaster NL, Ordnance Survey, Esri Japan, METI, Esri China (Hong Kong), OpenStreetMap contributors, and the GIS User Community.

We focused our study on the city of New Orleans, Louisiana, located in southeastern USA. (Figure 1). The city is home to 390,144 residents, and has a population density of 5255 people per km^2^ [16]. Median income in 2019 was estimated to be USD 41,604 [16]. Average precipitation is 1590 mm annually, and temperature averages range from 11.9 °C in the winter to 28.5 °C in the summer. New Orleans is one of the few locations in the USA, where *Ae. aegypti*, and *Ae. albopictus* are known to currently coexist [2].

Within New Orleans, we focused our study in cemeteries across the city. Cemeteries are ideal locations for the study of these species. They provide ample containers as larval rearing sites, as well as shelter and food sources for adults, both in terms of sugar sources from ornamental plants, and blood sources from cemetery visitors [17]. For research, they are ideal locations to study as they provide easier access to larvae samples, compared to sampling in private residences [17], and unlike discarded container habitats (e.g., trash and waste) they are often distributed equally across neighborhoods of different socioeconomic characteristics. Because vegetation was an important factor of interest, we first selected five cemeteries that had a mixture of trees and open spaces for comparison. In addition, we sampled two cemeteries that had been reported to have populations of *Ae. aegypti*, on previous studies [18]. The seven cemeteries selected were in the planning districts of Lakeview (Metairie and Holt cemeteries, New Orleans, LA, USA), Uptown (Carrolton cemetery, New Orleans, LA, USA), Central City (Lafayette no. 1 and Lafayette no. 2 cemeteries, New Orleans, LA, USA) and St. Roch (St. Roch and St. Vincent de Paul cemeteries, New Orleans, LA, USA). These cemeteries span a gradient of income levels above and below the median income for New Orleans (Table 1). Sampling was limited to areas of the cemetery within nearest to residential neighborhoods (~150 m) to ensure feeding sources for adult female mosquitos within their flight range.

### 2.2. Mosquito Larvae Collection

Sampling took place monthly between January 2020 and December 2020, with the exception of March 2020 due to COVID 19 stay at home orders. Two people inspected cemetery vases until larvae positive vases were found, both under the canopy of tree and under open space. If no larvae sample was found after 30 min. of searching a cemetery, the search concluded. Because of the size of the cemetery, this timeframe was considered sufficient to cover the entire cemetery by two people. The largest cemetery, Metairie, was sampled in the area closest to a residential neighborhood, and divided into three sections, all of which were inspected for a maximum of 30 min. On each sampling occasion, a maximum of six samples were collected in this larger cemetery, whereas a maximum of two samples (one open, one closed) were collected on other cemeteries. Water in the container, including larvae was mixed and collected entirely for analysis in the laboratory. Samples were stored in a cooler until larvae identification. Larvae were counted and identified to species using morphological keys [19]. During each sampling event, the sampled vase was emptied of contents and returned to its place. Therefore, some vases may have been sampled in more than one occasion. However, we considered each sample independent because contents were entirely removed between sampling events.

During the months of May to December 2020, a container survey was conducted on each sampling event, where all water-filled containers found with and without larvae during the 30-min search were counted and classified as larvae positive or larvae negative. This effort was meant to capture the number of vases inspected during our search period and the actual availability of larvae habitat (i.e., water-filled vases) on each of the cemeteries. We calculated a container index value or the percentage (%) of water holding containers with larvae [20] for these sampling months (Table 1). While our larvae sampling was destructive, we only limited our collection to one open and one closed canopy vase. Therefore, even if we found more than one larvae positive container, we did not collect more than two samples per cemetery per occasion to limit our interference with larvae habitat in subsequent sampling events.

### 2.3. Temporal Abiotic Factors

We characterized temperature and rainfall variability through the sampling year using Daymet: Daily Surface Weather Data, which provides data on a 1-km grid for North America [21]. Based on the sampling date for each month, we estimated rainfall and temperature in 1–4-week time lags. Climatic variables were estimated for each sampling date and for each cemetery, individually.

### 2.4. Cemetery Scale Habitat Characterization

We delimited a 200 m buffer around each cemetery using ArcGIS (ver. 10.8) to demark the flight range (~200 m) of *Aedes* species [22]. Within this buffer, we characterized UHI, vegetation cover, and median income. We used the UHI layer developed by the Trust for Public Lands [23]. This layer consists of a 30 m grid for cities in the U.S., depicting a heat severity index, ranging in values from 1 to 5, with 1 being slightly above the mean for the city and 5 being a significantly above the mean for the city [23]. The index was developed following the Jenks Natural Breaks classification method [24], and derived from Landsat 8 imagery band 10 (ground level thermal sensor) from summers 2018 and 2019. For vegetation cover, we used the USDA Forest Service percent tree canopy cover layer, a 30 m raster layer, calculated using a random forest regression algorithm and based on the National Agriculture Imagery Program (NAIP) aerial photographs [25,26]. Median income was determined using the American Community Survey 2017—5-year summary files [27] datasets.

### 2.5. Container Scale Habitat Characterization

For each larvae positive container sampled, we documented water temperature with a handheld thermometer, and estimated canopy cover with a spherical convex densitometer (Forestry Suppliers, Inc. Jackson, MS, USA). All vases consisted of approximately the same water volume (1 L or less), containers larger than this volume were rare, and were not considered, in order to standardize for this variable.

### 2.6. Statistical Analysis

The influence of temporal abiotic factors (temperature and rainfall variables with time lags) on larvae abundance per species were examined using quasipoisson regressions to account for over dispersion. Prior to the analyses, all variables were mean centered and scaled, except for the larvae response variables, which were treated as counts.

The relative importance of factors at the cemetery scale and the container scale was examined using a generalized linear mixed effects model (GLMM), using a negative binomial distribution to account for over dispersion. For the container scale characterization, samples were classified in terms of canopy cover above the container as either open (<60% canopy cover) or closed (>60% canopy cover), and transformed into a dummy variable (1: closed, 0: open).

**Figure 2:**
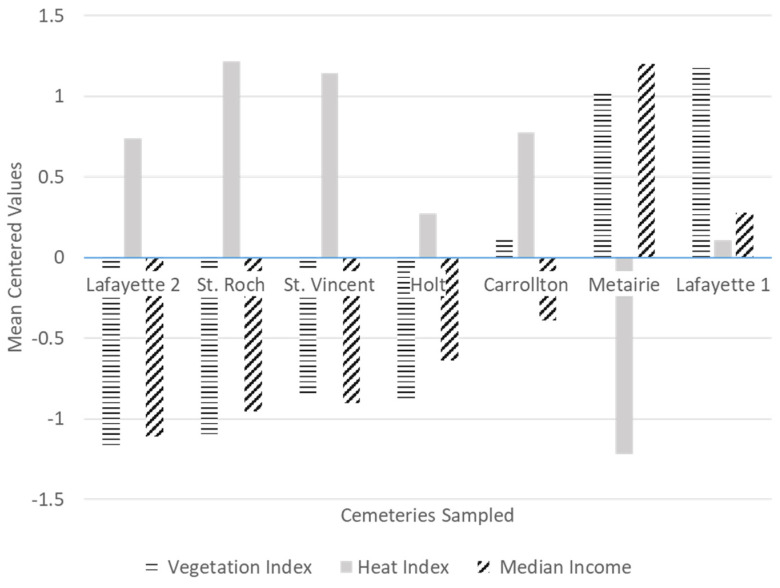
Cemeteries sampled depicted across a gradient of income level, heat index, and vegetation cover within a 200 m buffer around the sampled locations. Values represent the mean centered values for each of the variables.

The evaluated variables at the cemetery scale (200 m buffer), heat index, vegetation index and income, were converted into categories of high and low (1: high, 0: low) following the gradient illustrated in Figure 2 and Table 1. These three variables were highly correlated to each other, according to Pearson correlation coefficients of 0.90+ (Appendix A) and thus were examined separately. For the GLMMs, we added the variable cemetery as a random effects factor, to control for the influence of sampling on the same site on repeated occasions. The response variable for all models consisted on counts for each of the species.

All analyses were performed in R version 3.6.1 [28]. Quasipoisson regressions were conducted using the stats package in R. For the GLMM we used package lme4 [29]. We tested for overdispersion using package blmeco [30]. Some of our graphs were developed using the package ggplot2 [31].

## 3. Results

### 3.1. General Trends

We found a total of 87 larvae positive containers across all sampled cemeteries, 52 under an open canopy, and 35 under a closed canopy (Table 2). From these vases, we collected a total of 1611 mosquito larvae. The most abundant species was *Cx. quinquefasciatus* (*n* = 748), followed by *Ae. aegypti* (*n* = 559), and lastly *Ae. albopictus* (*n* = 304). We found more *Cx. quinquefasciatus* in open canopy (*n* = 535) than in closed canopy vases (*n* = 213). We found more *Ae. aegypti* in closed canopy (*n* = 319) than in open canopy vases (*n* = 240). *Ae. albopictus* were found in similar numbers in both open (*n* = 157) and closed canopy vases (*n* = 147). Water temperature was on average slightly lower for open (23.4 °C), versus closed canopy vases (24.4 °C), but maximum and minimum temperature ranges were wider for open canopy vases (max = 34.2 °C, min = 7.8 °C) versus closed canopy vases (max = 29.9 °C, min = 11.8 °C). The container index was highest in St. Vincent de Paul (0.41), followed by Lafayette 2 (0.34), Carrolton (0.08), Lafayette 1 (0.04), and lastly Metairie (0.04) (Table 1). Two cemeteries (St. Roch, Holt) could not be accessed in certain months, and therefore we did not include their data for the container index calculations. Coexistence among the species of interest was detected in 8 out of 87 vases samples (~9%) (Appendix B). The vases that had coexistence were located in Carrolton, Holt, Metairie, and St. Vincent de Paul cemeteries. For these vases, we observed a trend for a greater proportion of *Ae. albopictus* larvae in closed canopies, while the opposite was true for *Ae. aegypti*. This trend was not statistically tested, as there were so few of cases (four open canopy vases, four closed canopy vases). Coexistence in vases occurred only during peak mosquito season (May–October), and most often detected in the St. Vincent de Paul cemetery.

### 3.2. Temporal Abiotic Factors

**Figure 3:**
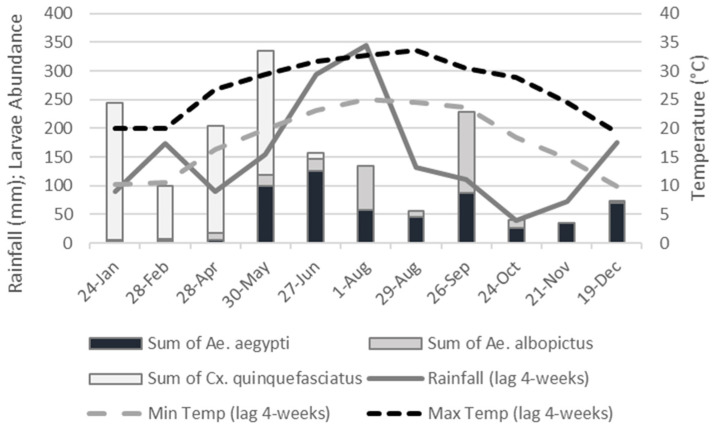
Temporal variability on temperature and rainfall through the sampling year (2020), and the relative abundances of the most abundant mosquito species. Climatic variables are represented in 4-week time lags, as this was the most closely related variable to the species of interest (Table 3).

Overall, temperature ranged from a minimum of 7.59 °C in December, to a maximum of 33.53 °C in August. Accumulated rainfall on a 4-week lag ranged from a minimum of 40 mm in October to a maximum of 345 mm in August. Detailed rainfall and temperature estimates for each sampling occasion are included in Appendix C, and a subset of these values are illustrated on Figure 3.

No significant relationships were found between *Ae. aegypti* and the climatic factors (Table 3). *Aedes albopictus* varied proportionally with maximum temperature with a 4-week time lag (Pr(>|t|) = 0.046), and to minimum temperature with 2–4-week time lags, (Pr(>|t|)<0.05, Table 3). No relationship was found between *Ae. albopictus* and rainfall moving averages. For *Cx. quinquefasciatus* a significant negative relationship was found with maximum temperature with 4-week time lag (Pr(>|t|) = 0.029). This species showed a negative relationship between minimum temperatures with 2–4 week time lags (Pr(>|t|< 0.05, Table 3). A negative relationship was found between *Cx. quinquefasciatus* and rainfall at the 1-week lag (Pr(>|t|) = 0.022) and the 4-week lag (Pr(>|t|) = 0.040).

Because *Cx. quinquefasciatus* numbers declined during the peak mosquito season, starting in May, and were reduced to zero by August (Figure 3), we considered this species to be temporally segregated from the other two species in the locations under study. Therefore, we excluded this species from subsequent analyses, as these focus on evaluating the spatial segregation patterns between species coinciding temporally in the sampled locations.

### 3.3. Cemetery Scale vs. Container Scale Factors

**Figure 4:**
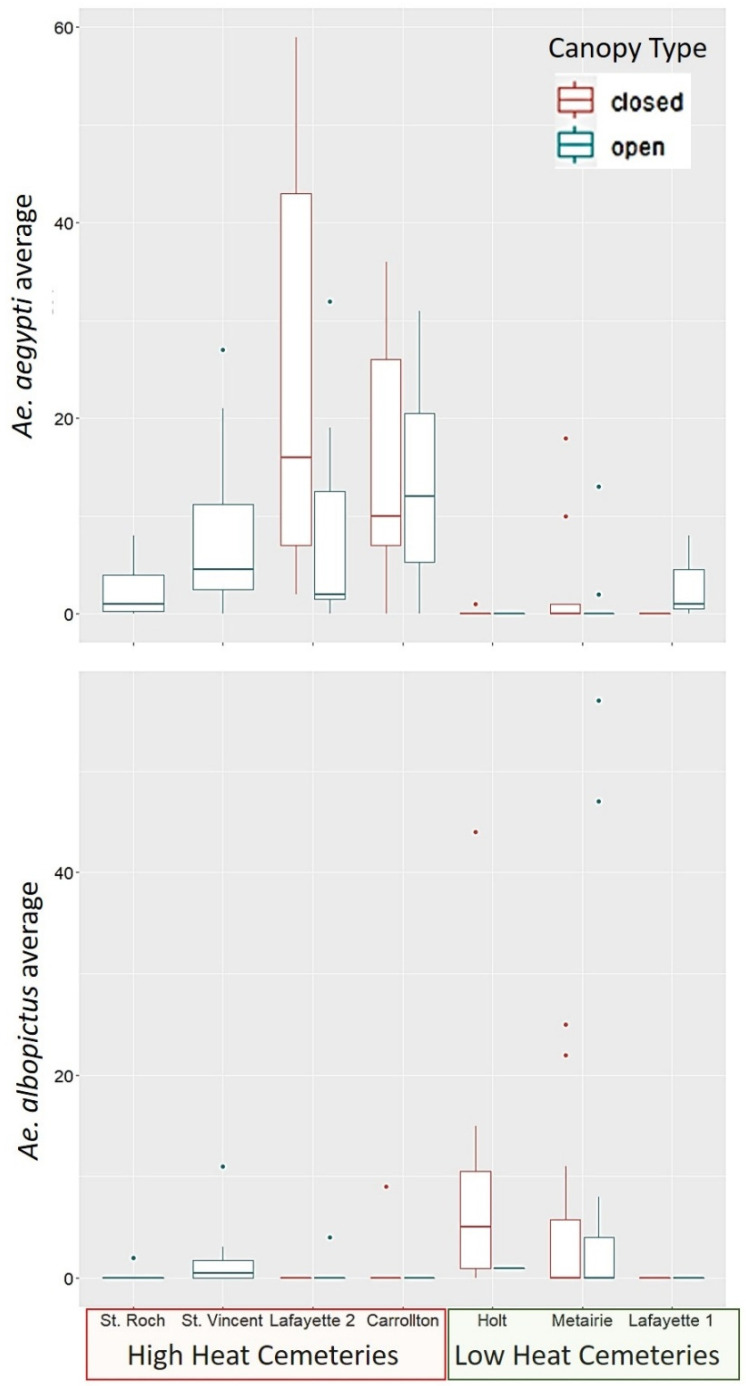
Relationship between mosquito abundance heat index (UHI) at the cemetery scale (high/low)), and canopy cover type at the container scale (closed/open). Other cemetery scale factors measured (vegetation index, median income) were not illustrated due to lack of significant relationship with the species collected with this study.

For *Ae. aegypti*, there was a significant effect of both the container canopy cover and heat index at the cemetery scale (Figure 4, Table 4). *Ae. aegypti* counts were positively associated to UHI (Pr(>|z| = 0.001, Table 4), and positively associated to canopy cover (Pr(>|z| = 0.0007, Table 4). Therefore, *Ae. aegypti* was most closely associated to vases within high heat cemeteries, and with closed canopy cover (Figure 4, Table 4). No other significant association was found between *Ae. aegypti* and the other cemetery scale variables measured.

While *Ae. albopictus* was more frequently collected in low heat cemeteries (Figure 4), there was no statistically significant relationship between UHI and this species (Table 4). There was no clear trend between canopy cover and *Ae. albopictus*, and no statistically significant relationship between the other cemetery scale variables (vegetation index, median income) and the species.

Random effects were examined using 97.5% confidence intervals. Confidence interval values did not overlap zero on any of the models tested, including cemetery and canopy scale for both species. The confidence intervals for random effects on the statistically significant models are as follows: *Ae. aegypti*/heat index (0.3346–1.5716); *Ae. aegypti*/canopy cover (0.9408–4.0412).

## 4. Discussion

Our study found that *Ae. aegypti* is a more abundant species than *Ae. albopictus* in the area sampled within the city of New Orleans (Figure 1). Within the cemeteries sampled, *Ae. aegypti* was most closely associated to locations with a high heat index at the cemetery scale (UHI). While the results were not statistically significant for *Ae. albopictus*, its distribution pattern shows the opposite trend, as the species was most often found in low heat cemeteries. These results suggest a spatial segregation pattern driven by heat tolerance of *Ae. aegypti*, as proposed in our hypotheses. These findings also support previous studies that classify *Ae. aegypti* as the more urban species, and *Ae. albopictus* as more associated to vegetated suburban/rural areas in Florida, USA [2,32,33], as well as several tropical cities [2,32,34,35,36].

We observed in our study that heat was negatively correlated to vegetation cover, and negatively correlated to median income (Appendix A). This suggests a disparity in terms of vegetation cover and heat exposure between low and high-income neighborhoods in New Orleans. More studies are needed to test this trend as we only sampled seven locations across the city. However, these findings are not surprising, as previous studies have reported unequal exposure to heat in low-income neighborhoods consistently across many cities of the USA [37,38]. Higher UHI exposure has been consistently shown to be linked to mortality and morbidity in humans [11], while access to green spaces may promote a variety of physical and mental health benefits [12]. Therefore, it would be important to further evaluate these apparent disparities to heat exposure and vegetation cover across socioeconomic divides in New Orleans.

Our findings show that high heat in particular exacerbates exposure to *Ae. aegypti*, which, according to previous studies, is a more effective disease vector than *Ae. albopictus* [14,15]. If we layer this finding with that of other studies suggesting how urban disrepair and abandonment create habitat for mosquitos and disproportionally affect low-income neighborhoods [5,6,7,8], we can see how there are multiple complex pathways by which poverty can be potentially linked to higher mosquito borne disease risk in our cities. Future studies should determine the role of mosquito habitat segregation in driving differences in relative disease risk within a city, accounting for differences in abundance and vector competence of the mosquito species present. Because of the tight correlation, we found that with UHI and income level in this study, we were not able to disentangle temperature related drivers, to container habitat availability drivers. The interaction between container habitat availability and UHI could present interesting dynamics for mosquito dispersal and therefore could be the basis for future research.

Contrary to our prediction, canopy cover at the container scale was not predictive of the presence of *Ae. albopictus*. Moreover, contrary to our prediction, canopy cover was positively associated with *Ae. aegypti*, in the locations were this species resided, which were high heat cemeteries, with low vegetation cover at the cemetery scale. Although *Ae. aegypti* tolerates higher temperatures, it makes sense that it would prefer to be under shaded conditions in these high heat localities, to experience less desiccation risk, and less extreme heat. Due to the lack of significant association found between *Ae. albopictus* to vegetation cover at any scale, our study does not support the hypothesis of habitat selection by this species is driven by the presence of trees and its competitive superiority at exploiting leaf litter. In laboratory studies, *Ae. albopictus* shows a superior ability to exploit leaf litter [39,40], and a preference to lay eggs in water infused with leaf detritus over water only containers [41]. However, there is no evidence in the literature that under natural conditions the species would preferentially select this food source, as it may be able to use other food sources available such as grass clippings and animal carcasses, which may offer more nutrient availability [39,40], and are not contingent on the presence of vegetation, or direct canopy cover.

The fact that *Ae. albopictus* was lower in abundance than *Ae. aegypti* in the sampled locations (which were all highly urbanized), and that the species was most often found in low heat cemeteries, suggests that temperature may be an important determinant of this species presence/absence across different parts of the city of New Orleans. Forecasting models have used the optimal temperature ranges for *Ae. aegypti* (21.3–34.0 °C) and *Ae. albopictus* (19.9–29.4 °C) to predict a decline in *Ae. albopictus* in the tropics in response to projected temperature increases due climate change [42]. It is possible that similar to climate driven temperature shifts, microscale variation in temperature ranges due UHI may also lead to temperature limits across a city’s landscape, shaping the segregation patterns of these two competing species.

Previous studies support the role of heat at limiting habitat suitability for *Ae. albopictus* within cities. A study conducted across urban to rural gradient in Athens, GA found that adult abundance of the species increased with temperature until a threshold of about 30 °C, after which adult abundance declined [43]. A similar study in Athens GA found that temperature was higher and relative humidity lower in urban vs. rural/suburban areas of the city, and that these microclimate differences resulted in lower *Ae. albopictus* larvae survival in the urban sites [44]. In Sao Paulo, where both species coexist, temperature increases have favored a greater expansion of *Ae. aegypti* across the city, relative to *Ae. albopictus* [45]. Accordingly, dengue infections have been positively correlated to UHI in Sao Paulo [46], while modelling studies using thermal optima for disease transmission suggest a higher thermal optimum for transmission of dengue by *Ae. aegypti* (29.1 (28.4–29.8)) than *Ae. albopictus* (26.4 (25.4–27.6); [47]). Together, these studies support the role of microscale variation in temperature as shaping the species segregation patterns in urban landscapes, and point towards a potential link of these segregation patterns to arboviral infection risk, which should be explored further.

We found few instances of coexistence between the species at the container scale (~9% of samples, Appendix B). The most frequent location for coexistence was in St. Vincent de Paul cemetery, which was a high heat cemetery dominated by *Ae. aegypti.* This cemetery is located near multiple water bodies that may promote a temperature mitigation effect in its surroundings that was not assessed by this study. More studies are needed to determine the factors that allow *Ae. albopictus* to inhabit this locality in spite of the high heat, and the potential role of proximity to water bodies as a mediating factor. Other instances where coexistence was detected at the vase level, shared the commonality of being closed canopy vases, and having *Ae. albopictus* at a higher relative abundance (Appendix B). These localities may be the focus of future studies to better assess the factors at the container scale that allow for coexistence, such as nutrient availability, and source and sink habitats.

The most abundant species collected in this study was *Cx. quinquefasciatus*, which was segregated temporally from the two *Aedes* species in cemeteries. A previous study in Tampa, Florida found a similar pattern, where *Cx. quinquefasciatus* switched habitat preference from cemeteries in the non-peak season, into other developed areas in the peak season, when the two *Aedes* species became more dominant [48]. The authors of the study suggest that coexistence among these competitors is enabled by spatial and seasonal segregation, and we provide further support for this finding in our study. We know from previous studies [49,50] and mosquito adult sampling in the city during 2020 (de Jesus, unpublished data) that *Cx. quinquefasciatus* is still abundant during the peak mosquito season in New Orleans. Therefore, the observed trend potentially reflects habitat switching to avoid competition with *Ae. albopictus*, which has been shown to be competitively superior to *Cx. quinquefasciatus* and other similar species such as *Culex coronator*, by laboratory studies testing interspecific competition under various resource levels [51,52].

Our study had several limitations. First, our monthly sampling was limited to seven cemeteries and one year of data. Longer-term studies at a larger scale would be necessary to fully understand spatiotemporal patterns of the mosquito species under study. Second, the temporal patterns reported on this study may have been affected by our sampling technique, which consisted of destructively sampling cemetery vases. This sampling strategy may have had an interference with the community assemblages in subsequent sampling events. However, since we limited our sampling to two cemetery vases per sampling occasion, while more were vases detected with water and larvae during our sampling, we think that our sampling interference was minimal. However, future studies may use non-destructive sampling strategies, such as oviposition traps to confirm our findings. Lastly, our sampling was limited to mosquito larvae, and we did not limit our collection to fourth instar larvae. Therefore, our survey does not fully reflect the outcomes of container scale competition or adult mosquito emergence, and more studies are needed to fully characterize the adult mosquito community assemblages across the city.

## 5. Conclusions

*Ae. aegypti*, and *Ae. albopictus* larvae show a pattern of spatial segregation in cemeteries of New Orleans. *Ae. aegypti* resides in high heat, low vegetation cemeteries, while *Ae. albopictus* was more often found in cemeteries with a lower heat index. Canopy cover over the container habitat in this highly urban environment is not predictive of *Ae. albopictus*, but it is predictive for *Ae. aegypti. Ae. aegypti* prefers to be under a shaded canopy in the high heat cemeteries where it resides. The qualities at the cemetery scale that are predictive of the habitat segregation patterns observed on this study (e.g., UHI) may be associated with dispersal limitation of *Ae. albopictus* under extreme heat conditions, but this hypothesis requires further evaluation. Our study suggests that high heat and low vegetation cover is characteristic of low-income neighborhoods in New Orleans, possibly highlighting multiple pathways for public health disparities in the city, including greater exposure to *Ae. aegypti*, which should be explored further.

## Figures and Tables

**Table 1:** Cemetery descriptive statistics (L) low classification, (H) high classification.

Cemeteries	Sampled Area (ha)	Median Income	Vegetation Index	Heat Index	Container Index (Avg. Se)
Lafayette 2	1.48	10,096	0.17	3.06	0.34 (0.11)
St. Roch	2.46	23,654	0.33	3.46	n/a
St. Vincent	2.31	28,482	0.75	3.40	0.41 (0.14)
Holt	2.64	51,765	0.69	2.67	n/a
Carrollton	3.79	73,750	2.45	3.09	0.08 (0.03)
Lafayette 1	2.21	132,750	4.32	2.53	0.04 (0.02)
Metairie	5.10	214,205	4.05	1.42	0.04 (0.009)

**Table 2:** Summary statistics describing container traits and species found.

Canopy Cover Category	Cemeteries	Vase Counts	Species Totals			Canopy Cover		Water Temperature			
			*Ae. albopictus*	*Ae. aegypti*	*Cx. quinquefasciatus*	Avg	Sd	Avg	Sd	Max	Min
Closed	Carrollton	5	9	79	70	83.83	13.86	24.32	3.89	27.8	17.9
	Holt	7	72	1	77	93.43	8.89	24.71	5.45	29.1	12.9
	Lafayette 1	2	0	0	14	99.61	0.55	26.25	0.07	26.3	26.2
	Lafayette 2	9	0	209	0	79.11	9.04	21.50	6.01	28	11.8
	Metairie	12	66	30	52	96.90	4.37	26.11	3.97	29.9	16.9
	Overall	35	147	319	213	89.92	11.12	24.40	4.91	29.9	11.8
Open	Carrollton	4	0	55	97	7.71	15.42	26.63	3.72	29	21.1
	Holt	1	1	0	0	39.42	0.00	13.30	0.00	13.3	13.3
	Lafayette 1	3	0	9	80	0.00	0.00	22.80	4.83	26.7	17.4
	Lafayette 2	7	4	62	0	0.00	0.00	22.23	8.20	29.3	10.6
	Metairie	21	132	15	358	6.33	14.35	24.69	5.71	31.4	14.3
	St. Roch	6	2	15	0	0.00	0.00	17.48	8.97	31.7	7.8
	St. Vincent de Paul	10	18	84	0	0.00	0.00	24.94	5.64	34.2	17.2
	Overall	52	157	240	535	3.91	11.44	23.39	6.65	34.2	7.8
	Grand Total	87	304	559	748						

**Table 3:** Regression results evaluating the role of temporal abiotic factors on mosquito assemblages during 2020.

	Week Lags	*Ae. aegypti*				*Ae. albopictus*				*Cx. quinquefasciatus*			
		Est.	S.E.	t	Pr(>|t|)	Est.	S.E.	t	Pr(>|t|)	Est.	S.E.	t	Pr(>|t|)
Maximum Temp.	1	0.25	0.23	1.1	0.28	0.25	0.34	0.72	0.47	−0.37	0.26	−1.41	0.16
	2	0.27	0.23	1.2	0.23	0.4	0.36	1.1	0.27	−0.43	0.26	−1.67	0.1
	3	0.26	0.23	1.15	0.26	0.72	0.41	1.76	0.08	−0.5	0.25	−1.96	0.05
	4	0.3	0.23	1.33	0.19	0.84	0.41	2.03	0.05	−0.58	0.26	−2.22	0.03
Minimum Temp.	1	0.33	0.23	1.4	0.16	0.74	0.42	1.76	0.08	−0.5	0.27	−1.9	0.06
	2	0.3	0.23	1.28	0.2	1	0.46	2.18	0.03	−0.5	0.26	−1.97	0.05
	3	0.29	0.23	1.27	0.21	1.08	0.45	2.43	0.02	−0.57	0.26	−2.17	0.03
	4	0.33	0.23	1.45	0.15	1.15	0.42	2.72	0.01	−0.68	0.27	−2.5	0.01
Moving Sum Rainfall	1	0.19	0.18	1.04	0.3	0.37	0.25	1.45	0.15	−1.31	0.56	−2.33	0.02
	2	0	0	−0.2	0.84	0.01	0	1.38	0.17	−0.01	0.01	−0.89	0.38
	3	0	0	0.9	0.37	0	0	0.29	0.77	−0.01	0	−1.55	0.12
	4	0	0	0.98	0.33	0	0	0.39	0.7	−0.01	0	−2.09	0.04

**Table 4:** Generalized linear mixed effects models for cemetery scale and container scale variables.

		Cemetery Scale			Container Scale
		Heat Index	Vegetation Index	Median Income	Canopy Cover
*Ae. aegypti*	Estimate	2.32	0.09	−1.44	0.71
	Std. Error	0.61	1.12	0.98	0.21
	Z Value	3.81	0.08	−1.48	3.4
	Pr(>|z|)	0.001	0.936	0.139	0.0007
*Ae. albopictus*	Estimate	−1.34	−0.23	1.25	0.06
	Std. Error	1.05	1.28	1.09	0.23
	Z Value	−1.28	−0.18	1.14	0.29
	Pr(>|z|)	0.201	0.857	0.253	0.77

## Data Availability

Data generated in this study are available upon request.

## Appendix A

**Table A1:** Table showing pearson correlation coefficients among explanatory variables. Variable definition: msmr = moving sum rainfall, maxt = maximum temperature, mint = minimum temperature. Each variable was estimated at 28-day, 21-day, 14-day, and 7-day lags. Temperature is in degrees Celsius. Rainfall is in inches. VegInd = Vegetation Index, HeatInd = Heat Index.

	vasetemp	vasecanopy	msmr7	msmr14	msmr21	msmr28	maxt7	maxt14	maxt21	maxt28	mint7	mint14	mint21	mint28	VegInd	HeatInd	Income
vasetemp	1.00	0.10	0.58	0.43	0.54	0.39	0.80	0.80	0.81	0.81	0.82	0.78	0.80	0.79	0.24	−0.19	0.22
vasecanopy	0.10	1.00	0.19	0.15	0.14	0.07	0.22	0.23	0.22	0.21	0.22	0.23	0.21	0.19	0.05	−0.12	0.05
msmr7	0.58	0.19	1.00	0.86	0.91	0.88	0.52	0.56	0.58	0.59	0.58	0.58	0.60	0.62	0.00	0.03	−0.02
msmr14	0.43	0.15	0.86	1.00	0.80	0.79	0.31	0.31	0.37	0.36	0.34	0.34	0.40	0.39	0.02	−0.03	0.02
msmr21	0.54	0.14	0.91	0.80	1.00	0.96	0.51	0.51	0.50	0.50	0.50	0.47	0.50	0.50	0.06	−0.01	0.02
msmr28	0.39	0.07	0.88	0.79	0.96	1.00	0.34	0.35	0.35	0.35	0.36	0.33	0.37	0.38	0.04	−0.01	0.01
maxt7	0.80	0.22	0.52	0.31	0.51	0.34	1.00	0.98	0.96	0.94	0.96	0.91	0.91	0.88	0.05	0.08	−0.03
maxt14	0.80	0.23	0.56	0.31	0.51	0.35	0.98	1.00	0.98	0.97	0.98	0.96	0.95	0.93	0.05	0.07	−0.02
maxt21	0.81	0.22	0.58	0.37	0.50	0.35	0.96	0.98	1.00	1.00	0.98	0.98	0.99	0.97	0.05	0.05	−0.01
maxt28	0.81	0.21	0.59	0.36	0.50	0.35	0.94	0.97	1.00	1.00	0.99	0.98	0.99	0.99	0.04	0.06	−0.02
mint7	0.82	0.22	0.58	0.34	0.50	0.36	0.96	0.98	0.98	0.99	1.00	0.98	0.98	0.97	0.05	0.05	−0.02
mint14	0.78	0.23	0.58	0.34	0.47	0.33	0.91	0.96	0.98	0.98	0.98	1.00	0.99	0.98	0.04	0.05	−0.02
mint21	0.80	0.21	0.60	0.40	0.50	0.37	0.91	0.95	0.99	0.99	0.98	0.99	1.00	0.99	0.05	0.03	0.00
mint28	0.79	0.19	0.62	0.39	0.50	0.38	0.88	0.93	0.97	0.99	0.97	0.98	0.99	1.00	0.04	0.04	−0.01
VegInd	0.24	0.05	0.00	0.02	0.06	0.04	0.05	0.05	0.05	0.04	0.05	0.04	0.05	0.04	1.00	−0.85	0.95
HeatInd	−0.19	−0.12	0.03	−0.03	−0.01	−0.01	0.08	0.07	0.05	0.06	0.05	0.05	0.03	0.04	−0.85	1.00	−0.95
Income	0.22	0.05	−0.02	0.02	0.02	0.01	−0.03	−0.02	−0.01	−0.02	−0.02	−0.02	0.00	−0.01	0.95	−0.95	1.00

## Appendix B

**Table A2:** Table showing individual samples in which coexistence among species within a container were detected, out of a total of 87 samples. Samples are labeled based on the cemetery where they were collected and characterized in terms of High (H) and Low (L) income, vegetation and heat index. Season in which coexistence was detected (Peak, Not-Peak) is listed, as well as counts for each of the species collected.

Cemetery	Vegetation Index	Heat Index	Canopy Type	Season	*Ae. albopictus*	*Ae. aegypti*	*Cx. quinquefasciatus*
Carrolton	H	H	closed	peak	9	7	0
Holt	L	L	closed	peak	15	1	0
Metaire	H	L	closed	peak	11	0	2
Metaire	H	L	closed	peak	4	1	0
St. Vincent	L	H	open	peak	2	21	0
St. Vincent	L	H	open	peak	3	4	0
St. Vincent	L	H	open	peak	1	2	0
St. Vincent	L	H	open	peak	11	2	0

## Appendix C

**Table A3:** Table listing meteorological estimates for each sampling date across the city. Data were derived from NASA DAYMET https://daymet.ornl.gov/ accessed on 14 September 2021. Variable definition: mint_ma = minimum temperature moving average, maxt_ma = maximum temperature moving average, msmr = moving sum of rainfall. Each variable was estimated at 28-day, 21-day, 14-day, and 7-day lags. Temperature is in degrees Celsius. Rainfall is in inches.

Date	mint28	mint21	mint14	mint7	maxt28	maxt21	maxt14	maxt7	msmr28	msmr21	msmr14	msmr7
24-Jan	10.16	10.2	11.46	7.8	19.97	19.83	20.07	16.74	90.6	72.05	51.2	15.34
28-Feb	10.51	10.73	9.91	7.94	20.02	19.96	18.75	17.48	174.59	96.02	77.64	1.37
28-Apr	16.35	16.8	15.76	16.93	26.78	26.98	26.62	28.09	89.42	87.46	81.08	7.81
30-May	19.82	20.83	22.27	22.69	29.32	29.49	30.81	30.84	154.78	136.9	50.22	37.89
27-Jun	23.16	23.19	22.91	23.15	31.69	31.73	32.27	31.62	293.85	270.94	92.44	92.44
1-Aug	25.05	25.28	25.11	24.47	32.76	32.78	31.88	31.23	345.35	301.57	206.13	160.97
29-Aug	24.49	24.47	24.36	24.9	33.53	33.32	32.98	32.12	131.73	118.48	67.15	54.39
26-Sep	23.69	23.1	22.72	21.18	30.4	29.52	27.67	25.27	110.5	73.11	59.36	36.34
24-Oct	18.48	19.05	19.55	19.04	28.82	28.68	28.91	28.26	40.13	33.85	23.44	21.69
21-Nov	14.79	14.02	15.71	12.45	24.48	24.28	24.99	24.02	72.03	17.41	10.84	0
19-Dec	9.83	8.13	7.59	8.1	19.34	17.58	17.36	15.94	175.24	85.23	21.27	19.63
